# Improving Learner-Computer Interaction through Intelligent Learning Material Delivery Using Instructional Design Modeling

**DOI:** 10.3390/e23060668

**Published:** 2021-05-26

**Authors:** Christos Troussas, Akrivi Krouska, Cleo Sgouropoulou

**Affiliations:** Department of Informatics and Computer Engineering, University of West Attica, 12243 Egaleo, Greece; akrouska@uniwa.gr (A.K.); csgouro@uniwa.gr (C.S.)

**Keywords:** adaptive learning material delivery, Component Display Theory, content-based filtering, Intelligent Tutoring Systems, Multiple-Criteria Decision Analysis, online learning, Weighted Sum Model

## Abstract

This paper describes an innovative and sophisticated approach for improving learner-computer interaction in the tutoring of Java programming through the delivery of adequate learning material to learners. To achieve this, an instructional theory and intelligent techniques are combined, namely the Component Display Theory along with content-based filtering and multiple-criteria decision analysis, with the intention of providing personalized learning material and thus, improving student interaction. Until now, the majority of the research efforts mainly focus on adapting the presentation of learning material based on students’ characteristics. As such, there is free space for researching issues like delivering the appropriate type of learning material, in order to maintain the pedagogical affordance of the educational software. The blending of instructional design theories and sophisticated techniques can offer a more personalized and adaptive learning experience to learners of computer programming. The paper presents a fully operating intelligent educational software. It merges pedagogical and technological approaches for sophisticated learning material delivery to students. Moreover, it was used by undergraduate university students to learn Java programming for a semester during the COVID-19 lockdown. The findings of the evaluation showed that the presented way for delivering the Java learning material surpassed other approaches incorporating merely instructional models or intelligent tools, in terms of satisfaction and knowledge acquisition.

## 1. Introduction

The field of education has undergone tremendous changes during the last decades. Indeed, the incorporation of technology means has altered the way with which learners gain knowledge. Furthermore, injecting artificial intelligence into learning technology systems can offer a more personalized learning experience to students [1]. With these developments, digital learning environments can be better tailored to learners’ personal and cognitive interests and preferences. Especially during the COVID-19 pandemic, the need for student-centric e-learning software is even more imperative, since face-to-face education has been fully replaced by digital learning [2]. Such urgent needs encompass the amelioration of the learning material and its adaptation to the learners so that it can be aligned to their special educational needs.

Adaptivity in the learning material embraces considerable pedagogical potential since it promotes the acquisition of knowledge in an environment that respects the cognitive characteristics of learners [3,4,5]. As such, learners can receive the educational material in a more accurate row and a more appropriate volume. According to the specific needs of each learner, the domain to be delivered can be tailored to these needs [6,7,8]. For instance, learners, who have poor knowledge on a topic, probably want to receive material sequentially with a high level of granularity; while learners with an advanced level of knowledge, probably want to see more superficially topics that are well known for them. During the COVID-19 pandemic, the experience of tutors in providing specific learning material to learners cannot be exploited due to the absence of face-to-face interaction, and thus, it is significant to use technological means to cover this issue.

The delivery of adaptive learning material to learners is a topic of growing interest by researchers in the related literature [9,10,11]. Several research efforts have been made towards adapting the domain knowledge to students in view of enhancing the pedagogical potential of the e-learning software. For example, researchers have explored the delivery of learning material to students by taking into account their preferences, namely their learning styles [12,13,14]. However, this adaptation is targeted mainly to the way of the domain knowledge presentation using learning styles (e.g., material for visual or auditory learners, etc.), and not to the type of activities delivered to the students. There have been several research efforts that explored the adaptivity of the domain knowledge to students in terms of their remembering skills [15,16,17], knowledge acquisition ability [18], perceiving facts [19,20,21], incentive and appeal [22,23,24], as well as the tutoring routes [25,26,27], domain knowledge style [28,29], external resources recommendation [30,31] and sequential teaching material [32,33,34]. Concerning the technology to achieve adaptation of the learning material, the researches in the afore-presented literature utilized discrete-time stochastic control process, machine learning, weighted conceptual diagrams, ontology learning and supervised and unsupervised techniques [35,36].

In conclusion, according to a review work of 2020 [37], the field of delivering adaptive learning material to learners is a quite under-researched area and thus, there is significant room for improvement in this direction. The motivation for this research derived from the need to explore the research area of adaptive domain knowledge. The majority of the research efforts inadequately explore the presentation of adaptive learning material. As such, there is free research space for researching issues like the type of learning material in order to maintain the pedagogical affordance of the system. Furthermore, the exploration of the utilization of novel technologies to enhance adaptation, like Multiple-Criteria Decision Analysis (MCDA) and Weighted Sum Model (WSM), triggered this research.

The novelty of the manuscript lies in organizing learning material based on Component Display Theory (CDT) and sophisticatedly adapting it to university students of Java programming language. The learning theory of CDT concentrates on the cognitive domain and deals only with the micro-level strategies for teaching a concept, principle, procedure, etc. providing detailed instructional prescriptions about how to support specific instructional outcomes with the appropriate material. As such, this theory in conjunction with the adaptation of the material can lead the effectiveness of learning to be improved. The learning material is stored in a digital repository being characterized by metadata, such as required knowledge level, misconceptions that it can correct, and CDT level. Using content-based filtering and MCDA, and specifically WSM, the system provides adequate learning material to learners. Content-based filtering is one of the common methods in recommending items similar to user characteristics; whereas, WSM was used since it is one of the most accurate methods for evaluating a number of alternatives in terms of a number of decision criteria. The weights of the WSM are determined by experts regarding the learning goals set.

## 2. Applying CDT to Support Learning

The domain knowledge of the presented system is the computer programming concepts, and specifically the programming language Java. It consists of 12 chapters, ranging from preliminary to advanced topics.

The modeling of the domain knowledge material is made using the Component Display Theory [38], tailored to determined performance levels. Hence, particular strategies are used orchestrating the learning of a single concept using components such as definitions, examples, and practice exercises [39]. CDT organizes learning into two dimensions:

1. Content dimension. It refers to the information that should be learned. Content ranges from simple forms, namely facts, to more complex ones, namely principles. The content in CDT includes the following types:
  - Facts—information proved to be true.
  - Concepts—general notions about a particular subject.
  - Procedures—a series of actions that should be performed in a certain manner in order to solve a problem or accomplish a goal.
  - Principles—the rules or assumptions that describe how something happens or works.
2. Performance dimension. It refers to the abilities of the learners to apply the content. Performance ranges from the simplest form, namely remembering, to an advanced form, namely finding. The three types of performance are:
  - Remembering—the memorization of the information and its recall.
  - Using—the ability of the learner to apply the information to a particular context.
  - Finding—the learner constructs new knowledge based on the content.
  - CDT determines four primary presentation forms:
  - Rules, an expository presentation of general concepts.
  - Examples, an expository presentation of cases related to a concept.
  - Recall, the inquisition of general concepts.
  - Practice, the inquisition of cases.

It also includes some secondary presentation forms, such as prerequisites (other concepts required to have been studied before), objectives (the learning goals intended to be attained), help (useful material for the better understanding of the concept), mnemonics (memory aids for remembering the concept) and feedback (analysis of the interaction between learning goals and components of the concept).

To decide the level of performance required for an area of content, the matrix is set up. In CDT, it can be concluded for each of the groups in the matrix that there is a mixture of primary and secondary presentation types that will offer the most effective and efficient acquisition of available skills and information. CDT specifies that when it includes both the required primary and secondary types, instruction is more effective. A complete instruction session must then consist of a goal, accompanied by a mixture of rules, examples, recall, practice, feedback, helps, and mnemonics relevant to the subject and learning task.

The theory is mainly intended for use by groups of students. Several components are given so that a wide range of learners can participate, but learners need only the components that function best with them to accomplish the instructional objectives (Table 1).

## 3. Adaptation of Learning Units

Adapting learning material to students’ needs is crucial for providing a more effective and efficient learning process. As such, the system, developed in this study, aims to improve student performance and engagement by selecting the most proper learning units based on student characteristics using content-based filtering and delivering them according to the learning goals set by employing WSM (Algorithm 1). 

The learner characteristics used in the developed algorithm concern learner cognitive level and skills, which are:

- Knowledge level (SP.KLe), emerged from the total score of student tests on a 100-point scale.
- Prior knowledge (SP.PKLe) on related domain concepts on a 100-point scale, as a result of the pre-test given to students at the beginning of the course.
- Degree of misconceptions (SP.DoM), namely a degree ranging from 0 to 1 indicating the type of mistakes the student usually does in the tests. Since the course taught is a programming language, the possible mistakes that can be done are syntactic or logical. The nearer the degree of SP.DoM is to zero, the more often a student does syntactic errors; whereas, the nearer this degree is to 1, the more often the student makes logical mistakes.
- Student performance on a 100-point scale in CDT levels, arisen from the answers on question items of the tests given; namely, student performance on the following levels:
  ○ Use-Concept (SP.UCon)
  ○ Use-Procedure (SP.UPro)
  ○ Use-Principle (SP.UPri)
  ○ Find-Concept (SP.FCon)
  ○ Find-Procedure (SP.FPro)
  ○ Find-Principle (SP.FPri)
  ○ Remember-Fact (SP.ReFa)
  ○ Remember-Concept (SP.ReCon)
  ○ Remember-Procedure (SP.RePro)
  ○ Remember-Principle (SP.RePri)

The values of the above variables, except SP.PKLe, are defined dynamically by the adaptive educational system at each interaction of the learner with the system.

Regarding the learning units, the instructors have to define the following metadata when they insert them into the repository, essential for the content adaptation:

- The knowledge level that the learning units concern (LU.KL), stated as a score on a 100-point scale.
- The learner’s previous knowledge (LU.PKLe), expressed on a 100-point scale, which is a prerequisite for studying the learning unit.
- The degree of misconceptions (LU.DoM), a number between 0 to 1, indicating whether the particular learning unit is suitable for learners that make syntax (near to 0) or logic mistakes (near to 1).
- The degree, stated as performance on a 100-point scale, that the particular learning unit is suitable for each CDT level. The CDT levels are the same with those described in student performance; as such, the following names are given for the CDT levels referred to learning units: LU.UCon, LU.UPro, LU.UPri, LU.FCon, LU.FPro, LU.FPri, LU.ReFa, LU.ReCon, LU.RePro, LU.RePri.

**Algorithm 1. Process for intelligent selection and delivery of learning units.**
h: the number of features (student characteristics/metadata), namely 13. S: students enrolled into the course. S(i): the vector with the values of the 13 characteristics of student i. LU: the set of learning units that are included into the repository. LU(j): the vector with the values of the 13 metadata of learning unit j. RU ⊆ LU: the set of recommended learning units based on student characteristics. RU(k): the vector with the values of the 13 metadata of recommended learning unit k. n: the number of learning units in RU. D: the set of calculated distances for each learning unit, used in content-based filtering. w: the vector with the 13 relative weights of importance of the criterion that is associated with the corresponding metadata.

1. Experts define the values of n and w.
2. The student i searches for learning material.
3. The system applies the content-based filtering method in LU.
  - Calculate distance metric based on student characteristics and learning units metadata.
    (1) $$D\left(i,\;j\right)=\sqrt{\;\sum_{c=1}^{h}{\left(S_{i}\left(c\right)-LU_{j}\left(c\right)\right)}^{2}}$$
  - Sort D in ascending order.
  - Set in RU the top n learning units of D.
4. The system applies the WSM method in RU.
  (a) Normalize the values of learning units according to beneficial and non-beneficial attributes.
    For beneficial attributes, where g: LU.KL, LU.PKLe and CDT levels:
    (2) $$RU_{k}\left(g\right)=RU_{k}\left(g\right)/\max(RU\left(g)\right)$$
    For non-beneficial attributes, where g: LU.DoM:
    (3) $$RU_{k}\left(g\right)=\;\min\left(RU\left(g\right)\right)/RU_{k}\left(g\right)$$
  (b) Calculate the weighted scores.
    (4) $$RU\left(k\right)=\overset{h}{\underset{c=1}{\sum }}w\left(c\right)*RU\left(k\right)$$
  (c) Sort RU in descending order based on the WSM scores (if two or more learning units have the same WSM score, then they are sorted randomly).
5. Return the content of RU to the student.

## 4. Example of Operation

For a better understanding of the algorithm, a representative example is provided in Table 2. In this case, the experts set the WSM weights giving higher values in student knowledge level and misconceptions, as well as in complex/advanced dimensions of CDT. The reason why the weights were defined in this way is that the learning goal is for students to achieve higher-order cognitive skills. The student S1, selected for this case, has intermediate performance and tends to make serious mistakes, as his/her degree of misconception is high. Moreover, s/he has lower performance in complex/advances dimensions of CDT. In this example of operation, a sample of eight learning units referred to several cognitive levels is chosen; from them, the 5 more suitable for the student, regarding his/her profile and the learning goals set, are delivered.

In the first stage, content-based filtering is applied to sample data based on the formula shown in step 3a of the algorithm. As such, for each value of the feature vector of each learning unit, the distance from the corresponding value of the student’s feature vector is calculated, and the total distance emerges from the square root of their sum (Table 3). According to the distance values, the order of the 5 learning units selected, as being closer to student profile, is LU_3_, LU_8_, LU_5_, LU_7,_ and LU_4_ (Table 3). 

In the next stage, WSM is employed in the selected five learning units in order to be sorted based on the learning goals defined by the WSM weights. Firstly, the values of the feature vector of each learning unit in Table 2 should be normalized following the rule described in step 4a of the algorithm. As such, the maximum value is detected for the beneficial attributes and the minimum value for the non-beneficial attributes (Table 4). It should be noted that the non-beneficial attributes are those in which minimum values are desired. Hence, the only non-beneficial attribute in the presented approach is the DoM (3rd column in the feature vector). 

Afterward, the values are normalized using the formulation x/x_max_ for beneficial attributes, and x_min_/x for non-beneficial attributes (Table 5).

Finally, the WSM scores are calculated based on the formula presented in step 4b of the algorithm, i.e., each normalized value of Table 5 is multiplied with the respective weight of Table 2, and their sum corresponds to the WSM score (Table 6). Based on this score, the rank of the five learning units is redefined (Table 6). As a result, the learning units are delivered in the following order: LU_4_, LU_3_, LU_7_, LU_8,_ and LU_5_.

## 5. Evaluation Procedure

The phase of evaluation plays a crucial role in the life cycle of educational software; this phase identifies if the system meets the initial requirements and objectives. The phase of the evaluation is oriented to the assessment of the delivery of learning units to students.

### 5.1. Materials and Methods

The evaluation was conducted in a computer science department of a public university in the capital city of the country. The population consists of 120 undergraduate students attending the mandatory course of the programming language Java. All the students are in the first year of their studies and, as such, they have approximately the same age and educational level. The whole process of the evaluation lasted a semester, during the COVID-19 lockdown. It needs to be noted that the course was delivered in a fully online and synchronous way, while the students used the system all this time to support their studies. The students showed great interest in the system and were supported by the two course lecturers when needed.

The students were divided into three equal groups (Groups A, B, C) by the lecturers (40 students in each group). Towards keeping a high level of quality in the evaluation process, the lecturers divided students into groups very carefully respecting their characteristics (Table 7).

During the evaluation process, learners in Group A (experimental group) have used a system that integrated the presented approach, namely the delivery of adaptive learning material using CDT and WSM, while students in the control groups (Groups B, C) have used two conventional versions of the aforementioned system having the same user interface. Specifically, Group B has used a system that incorporated only the CDT theory for learning material delivery and Group C used a system that incorporated only WSM for learning material delivery.

One-way ANOVA was used to measure the satisfaction of learners of three groups concerning the delivery of adaptive learning material. Two control groups were used in order to investigate how successful the delivery of adaptive learning material is. There will be a comparison between the intelligence of the presented method (Group A) and the models of CDT (incorporated in the system used by Group B) and WSM (incorporated in the system used by Group C), individually. As such, at the end of the evaluation phase, the three groups were asked to reply to the following questions based on the Likert scale, namely from ‘Very much’ (10) to ‘Not at all’ (0):

- Was the learning material based on the level of your knowledge? (Q1)
- Was the learning material based on the level of your CDT level? (Q2)
- Did the learning material help you advance your performance? (Q3)

The ANOVA test was applied to students’ answers regarding their satisfaction with the proposed learning material; hence, the null hypothesis is that there is no difference in the delivery of adaptive learning material between the experimental and control groups.

### 5.2. Results and Discussion

The results reveal that the null hypothesis is rejected for all three questions, since F_Q1_ = 28.56 > F-crit_Q1_ = 3.07, F_Q2_ = 30.60 > F-crit_Q2_ = 3.07 and F_Q3_ = 28.08 > F-crit_Q3_ = 3.07 (Table 8 and Table 9). This fact shows that the means of the three groups are not all equal. However, in order to determine the group that surpasses the others, the calculation of the Least Significance Difference (LSD) is needed. In our case, the LSD gets the value of 0.56. Comparing LSD with the difference of the means of Group A-Group B and Group A-Group C for each question, the results show that the differences are greater than it. In particular, in Q1, the difference of the means of Group A and Group B is 1.475; while this of Group A and Group C is 2.35 (Q1: diff_AB_ = 1.475 > 0.56, diff_AC_ = 2.35 > 0.56). Hence, the presented system provides a more appropriate method for delivering learning units according to student knowledge than the other systems. Regarding Q2, the difference of the means of Group A and Group B is 0.8; while this of Group A and Group C is 2.32 (Q2: diff_AB_ = 0.8 > 0.56, diff_AC_ = 2.32 > 0.56). This fact indicates that the learning material provided by the presented system corresponds better to student CDT level compared to the simple approaches. Finally, concerning Q3, the difference of the means of Group A and Group B is 1.23; while this of Group A and Group C is 2.12 (Q3: diff_AB_ = 1.23 > 0.56, diff_AC_ = 2.12 > 0.56). As such, the presented system has a more positive effect on student performance than the other two systems. 

To sum up, the recommendation of learning material incorporating content-based filtering and WSM outperforms the simple approaches where only one method is used. More specifically, the content-based filtering ensures the selection of the most suitable material for student profile, while the WSM reorders the selected learning units to better fit the learning goals set. The findings support the effectiveness and efficiency of the presented system regarding the successful delivery of content based on students’ knowledge level and CDT level, as well as regarding the improvement of learning outcomes.

## 6. Conclusions and Future Work

In this paper, an innovative approach for the delivery of adequate learning material to students is presented. To achieve this, the approach encompasses the combination of an instructional design theory and intelligent techniques. More specifically, CDT is used for organizing the learning material. It functions in conjunction with the content-based filtering and MCDA, and specifically WSM, and as such, adequate learning material is delivered to learners.

As a testbed for this research, a prototype intelligent tutoring system has been developed. The system was used for a semester for the teaching of Java programming to university students during the COVID-19 lockdown. The system has been evaluated with promising results. Specifically, it was compared to two conventional versions, incorporating merely instructional models or intelligent tools, and it was found that it surpasses them in terms of satisfaction and knowledge acquisition.

Limitations of this work include that the WSM weights of the presented model are set by the experts according to the learning goals of the students. However, this process could be automated and the weights could be set dynamically in order to take into account the learning progress and performance of the students.

Future steps include the utilization of other intelligent techniques, like artificial neural networks, in order to investigate the effectiveness of the delivery of learning material.

## Figures and Tables

**Table 1:** Applying CDT to Java learning.

	Fact	Concept	Procedure	Principle
Use	-	Identify or classify Java objects, methods, etc.	Demonstrate programming procedures	Explain why a program is running or predict the output of the program
Find	-	State or define terms, e.g., class, object, etc.	State steps	State relationship inside a program
Remember	Recall or reorganize parts of the program	Recall or reorganize definitions	Recall or reorganize steps to build a program	Recall or reorganize principles of a program

**Table 2:** Sample Data: Feature Vectors of WSM Weights, A Student & 8 Learning Units.

	Feature Vector *
W	0.15 0.09 0.15 0.05 0.05 0.10 0.05 0.05 0.09 0.05 0.05 0.06 0.06
S_1_	67 60 0.78 65 63 58 68 64 61 71 67 66 59
LU_1_	90 85 0.10 90 85 80 90 85 80 90 90 85 85
LU_2_	80 80 0.30 70 70 70 80 80 80 80 80 80 80
LU_3_	70 60 0.50 70 65 60 70 65 60 70 65 65 60
LU_4_	75 65 0.40 70 70 65 75 75 70 80 75 70 70
LU_5_	65 60 0.70 60 60 60 60 60 60 65 65 65 65
LU_6_	55 45 0.85 50 50 50 55 55 50 55 55 55 50
LU_7_	70 50 0.70 75 70 65 75 70 65 75 70 70 65
LU_8_	65 55 0.60 65 65 60 70 65 60 75 70 65 65

* {KLe, PKLe, DoM, UCon, UPro, UPri, FCon, FPro, FPri, ReFa, ReCon, RePro, RePri}.

**Table 3:** Applying content-based filtering in sample data.

	Calculation of (S_i_(c)—LU_j_(c))^2^													Sum	Distance	Order
LU_1_	529	625	0.46	625	484	484	484	441	361	361	529	361	676	5960.46	77.21	8
LU_2_	169	400	0.23	25	49	144	144	256	361	81	169	196	441	2435.23	49.35	7
**LU_3_**	9	0	0.08	25	4	4	4	1	1	1	4	1	1	55.08	7.42	**1**
**LU_4_**	64	25	0.14	25	49	49	49	121	81	81	64	16	121	745.14	27.29	**5**
**LU_5_**	4	0	0.006	25	9	4	64	16	1	36	4	1	36	200.006	14.14	**3**
LU_6_	144	225	0.005	225	169	64	169	81	121	256	144	121	81	1800.005	42.43	6
**LU_7_**	9	100	0.006	100	49	49	49	36	16	16	9	16	36	485.006	22.02	**4**
**LU_8_**	4	25	0.03	0	4	4	4	1	1	16	9	1	36	105.03	10.25	**2**

**Table 4:** Indicating the max value for beneficial features and min value for non-beneficial features.

	Feature Vector *												
LU_3_	70	60	0.50	70	65	60	70	65	60	70	65	65	60
LU_8_	65	55	0.60	65	65	60	70	65	60	75	70	65	65
LU_5_	65	60	0.70	60	60	60	60	60	60	65	65	65	65
LU_7_	70	50	0.70	**75** **(max)**	**70** **(max)**	**65** **(max)**	**75** **(max)**	70	65	75	70	**70** **(max)**	65
LU_4_	**75** **(max)**	**65** **(max)**	**0.40** **(min)**	70	70	65	75	**75** **(max)**	**70** **(max)**	**80** **(max)**	**75** **(max)**	70	**70** **(max)**

* {KLe, PKLe, DoM, UCon, UPro, UPri, FCon, FPro, FPri, ReFa, ReCon, RePro, RePri}.

**Table 5:** Normalization of values.

	Feature Vector *												
LU_3_	70/75	60/65	0.40/0.50	70/75	65/70	60/65	70/75	65/75	60/70	70/80	65/75	65/70	60/70
LU_8_	65/75	55/65	0.40/0.60	65/75	65/70	60/65	70/75	65/75	60/70	75/80	70/75	65/70	65/70
LU_5_	65/75	60/65	0.40/0.70	60/75	60/70	60/65	60/75	60/75	60/70	65/80	65/75	65/70	65/70
LU_7_	70/75	50/65	0.40/0.70	75/75	70/70	65/65	75/75	70/75	65/70	75/80	70/75	70/70	65/70
LU_4_	75/75	65/65	0.40/0.40	70/75	70/70	65/65	75/75	75/75	70/70	80/80	75/75	70/70	70/70

* {KLe, PKLe, DoM, UCon, UPro, UPri, FCon, FPro, FPri, ReFa, ReCon, RePro, RePri}.

**Table 6:** Applying WSM in sample data.

	Feature Vector *													WSM	Rank
LU_3_	0.933 × 0.15	0.923 × 0.09	0.800 × 0.15	0.933 × 0.05	0.929 × 0.05	0.923 × 0.10	0.933 × 0.05	0.867 × 0.05	0.857 × 0.09	0.875 × 0.05	0.867 × 0.05	0.929 × 0.06	0.857 × 0.06	0.8898	2
LU_8_	0.867 × 0.15	0.864 × 0.09	0.667 × 0.15	0.867 × 0.05	0.929 × 0.05	0.923 × 0.10	0.933 × 0.05	0.867 × 0.05	0.857 × 0.09	0.938 × 0.05	0.933 × 0.05	0.929 × 0.06	0.929 × 0.06	0.8603	4
LU_5_	0.867 × 0.15	0.923 × 0.09	0.571 × 0.15	0.800 × 0.05	0.857 × 0.05	0.923 × 0.10	0.800 × 0.05	0.800 × 0.05	0.857 × 0.09	0.812 × 0.05	0.867 × 0.05	0.929 × 0.06	0.929 × 0.06	0.8265	5
LU_7_	0.933 × 0.15	0.769 × 0.09	0.571 × 0.15	1 × 0.05	1 × 0.05	1 × 0.10	1 × 0.05	0.933 × 0.05	0.929 × 0.09	0.938 × 0.05	0.933 × 0.05	1 × 0.06	0.929 × 0.06	0.8844	3
LU_4_	1 × 0.15	1 × 0.09	1 × 0.15	0.933 × 0.05	1 × 0.05	1 × 0.10	1 × 0.05	1 × 0.05	1 × 0.09	1 × 0.05	1 × 0.05	1 × 0.06	1 × 0.06	0.9967	1

* {KLe, PKLe, DoM, UCon, UPro, UPri, FCon, FPro, FPri, ReFa, ReCon, RePro, RePri}.

**Table 7:** Students’ characteristics.

Features	Group A	Group B	Group C
Average age	17.9	18.2	18.1
Sex	18 females 22 males	19 females 21 males	20 females 20 males
Demographics	Equivalent number of urban students and those of rural descent.		
Technology knowledge	Advanced experience in the use of technology.		
Previous knowledge	All students passed the national exams with similar grades in order to be admitted to the university.		
Motivation	All students attended the course of Java programming and expected a high grade to be attained.		

**Table 8:** ANOVA analysis of students’ feedback—Part I.

Quest.	Group	Count	Sum	Mean	Variance
Q1	Group A	40	331	8.275	1.49
	Group B	40	272	6.8	1.81
	Group C	40	237	5.925	2.64
Q2	Group A	40	332	8.3	1.70
	Group B	40	300	7.5	1.79
	Group C	40	239	5.98	1.97
Q3	Group A	40	338	8.45	1.13
	Group B	40	289	7.22	1.61
	Group C	40	253	6.33	2.12

**Table 9:** ANOVA analysis of students’ feedback—Part II.

Quest.	Source of Variation	SS	df	MS	F	P	F-Crit
Q1	Between Groups	112.85	2	56.43	28.56	7.93 × 10^−11^	3.07
	Within Groups	231.15	117	1.98			
	Total	344	119				
Q2	Between Groups	111.62	2	55.81	30.60	2.04 × 10^−11^	3.07
	Within Groups	213.38	117	1.82			
	Total	325	119				
Q3	Between Groups	91.02	2	45.51	28.08	1.1 × 10^−10^	3.07
	Within Groups	189.65	117	1.62			
	Total	280.67	119				

## Data Availability

Data available on request.
